# Implementation of the early-onset sepsis calculator in a tertiary care NICU

**DOI:** 10.1017/ash.2026.10363

**Published:** 2026-04-10

**Authors:** Walid Hussain, Deborah Bondi, Pooja Shah, Elzbieta Kalata, Kelli Skvarenina, Simon Parzen-Johnson

**Affiliations:** 1 Pediatrics, University of Chicago Hospital: The University of Chicago Medicine, Chicago, USA; 2 Pharmacy, University of Chicago Hospital: The University of Chicago Medicine, Chicago, USA

## Abstract

**Objective:**

Early-onset sepsis (EOS) is a significant cause of neonatal morbidity and mortality. Due to fear of missing cases, many newborns are unnecessarily exposed to antibiotics. We implemented the neonatal EOS calculator to reduce over-utilization of antibiotics and decrease costs.

**Design:**

Quality improvement study.

**Setting:**

Neonatal Intensive Care Unit (NICU).

**Patients:**

Infants born at ≥34 weeks’ gestation were divided between two periods: the pre-EOS calculator time frame and the post-EOS calculator time frame.

**Intervention:**

We changed our EOS evaluation for inborn infants by implementing the EOS-calculator and decreasing “rule-out sepsis” time frame from 48 to 36 hours for infants started on antibiotics.

**Results:**

1,306 infants, with similar demographics, were included: 814 in pre-EOS calculator time frame and 492 in post-EOS calculator time frame. Following our interventions, the percentage of NICU admissions ≥34 weeks’ gestation started on antibiotics decreased from 62% to 51% (*P* < .01). In the chorioamnionitis subgroup, antibiotic starts decreased by 50% (*P* < .01). There was a reduction in days of therapy per 1,000 NICU (168 vs 110, *P* < .01) and total (93 vs 57, *P* < .01) patient days. Fewer patients had blood cultures drawn (84% vs 67%, *P* < .01) with a decrease in infants treated for culture-negative sepsis (7% vs 3%, *P* < .01). NICU and hospital length of stay reduced by 1 day (*P* < .01), equivalent to a savings of $916,000 to $1.84 million per 1,000 NICU patients in costs and savings of $5.82 million to $12.5 million per 1,000 NICU patients in charges.

**Conclusions:**

Antibiotic usage significantly decreased, with substantial savings after implementation of the EOS calculator, without significant negative effects.

## Introduction

Newborn infants are at risk of early-onset sepsis (EOS), which is defined as a positive blood or cerebrospinal culture within 72 hours of birth, and it is a significant cause of neonatal morbidity and mortality.^1^ While EOS can affect all newborns, it disproportionally affects premature infants, with a 10-fold higher rate compared to term neonates.^2^ The overall incidence of EOS among term and late-preterm infants has declined significantly due to widespread use of intrapartum antibiotic prophylaxis directed at group *B Streptococcus*.^3,4^ Evaluation for EOS is common in the neonatal intensive care unit (NICU). For infants who are clearly ill, the decision to proceed is straightforward. However, many infants exhibit only minor abnormalities while appearing otherwise well, making the decision less clear. Most laboratory markers, including complete blood counts with differential, have been shown to be neither specific nor sensitive for diagnosis.^5,6^ In the setting of decreasing incidence of EOS amongst low-risk term and late-preterm infants, many infants are unnecessarily exposed to antibiotics due to fears of missing this diagnosis with high mortality.

Empiric antibiotics for suspected EOS is the main contributor to antibiotics early in life,^7^ with a high number of infants treated categorized as low-risk. Under previous recommendations, including the Center for Disease Control guidelines, the number of infants treated with empiric antibiotics was ∼ 200-fold higher than the incidence of EOS.^8^ Estimates suggest that, using these guidelines, 60–1,400 non-infected neonates would receive empiric antibiotics per single case of culture-proven EOS in clinically stable infants born to mothers with chorioamnionitis.^9^ Historically, most NICUs used a 48-hour “rule-out sepsis” course of empiric antibiotics if a culture remained negative, although the majority of bacteria grow within 24 hours.^10,11^ There is also a propensity to treat infants with longer courses of antibiotics (5–7 d) despite negative cultures, often referred to as “culture-negative sepsis” or “clinical sepsis.”^12^

Antibiotic exposure early in life is associated with both short-term and long-term risks. There are negative effects that are difficult to measure, like neonatal pain and parental anxiety, and more quantifiable effects such as unnecessary admissions to the NICU, higher healthcare costs, and interruption of maternal bonding and breastfeeding.^8^ Early life antibiotic exposure is also associated with necrotizing enterocolitis and death.^13,14^ Unnecessary antibiotics increase the risk of antibiotic resistance and the development of future childhood diseases, including asthma, allergic disorders, obesity, and autoimmune disorders.^15–21^ Lastly, there is evolving evidence that antibiotic exposure alters the developing neonatal microbiome, as well as brain development and function.^22,23^ The disruption of the microbiome is also associated with the development of other infections, including late-onset sepsis, with the predominant organisms being invasive fungal infection, as well as staph species to a lesser extent.^13^

Based on the evolving data regarding negative consequences of unnecessary antibiotics, many institutions have tried to decrease their utilization of antibiotics in newborn infants with the adoption of the Kaiser Permanente neonatal EOS calculator (2017).^24^ The tool was developed using a case-control design including a cohort of over 600,000 neonates. It is a multivariate tool which factors in prior probability based on the rate of sepsis in the study population that helps determine EOS risk based on several key maternal and neonatal risk factors. Maternal factors include temperature, group *B Streptococcus* status, intrapartum antibiotics, and rupture of membrane duration, while neonatal risk factors including gestational age and clinical status.

## Methods

### Setting and context

This single-center quality improvement project followed the SQUIRE 2.0 guidelines in all aspects of the project.^25^ The study was conducted at the University of Chicago Medicine Comer Children’s Hospital Level IV NICU, a 71-bed unit. The study population included all inborn infants born at 34 weeks’ gestation or greater, including those born to birthing parents diagnosed with chorioamnionitis. This project received a formal determination of quality improvement status according to institutional policy at University of Chicago Medicine and was therefore deemed not human subjects research and not reviewed by the Institutional Review Board. Prior to this study, any neonate requiring antibiotics was admitted to the NICU, and all infants born to a birthing parent diagnosed with chorioamnionitis received empiric antibiotics and NICU admission regardless of clinical status. Infants with major congenital anomalies or who died within 7 days of life were excluded.

**Figure 1.**
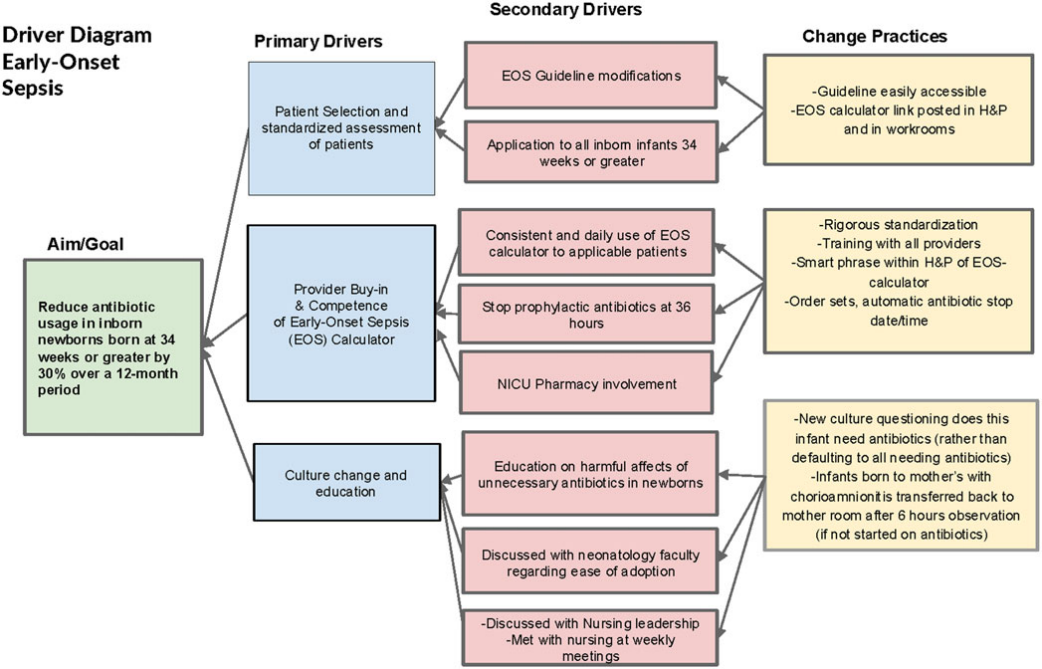
Early-onset sepsis key driver diagram.

Data were collected from June 2021 through November 2022 (pre-EOS calculator period). This baseline data informed staff education and guided changes to promote antibiotic stewardship, support maternal-infant bonding, and reduce hospital and patient costs. The multidisciplinary team—comprising neonatologists, NICU pharmacists, neonatal fellows, nurse practitioners, nurses, and the pediatric antimicrobial stewardship team—reviewed the data and set a SMART goal of reducing antibiotic use in inborn newborns 34 weeks or greater by 30% over 12 months, with key drivers shown in Figure 1.

### Intervention

After reviewing and evaluating the data, the EOS guideline was updated in February of 2023. The main changes were incorporating the Kaiser Permanente neonatal EOS calculator for all infants born at 34 weeks’ gestation or greater to estimate EOS risk and shortening empiric “rule-out sepsis” antibiotic duration from 48 to 36 hours. The goal was to safely reduce both the number of infants receiving antibiotics and the duration of exposure, thereby lowering costs. Retrospective review showed all culture-positive EOS cases grew within 24 hours, except one at 24.5 hours. The EOS incidence over the previous three years (∼1/1,000 live births) was used in the calculator. A washout period from December 2022 to January 2023 allowed presentation of baseline data and education of staff on the guideline changes and EOS calculator. Project champions assisted with training rotating providers, and an Epic SmartPhrase was embedded in the NICU history and physical template for documentation. Champions attended nursing meetings and huddles and presented at Labor & Delivery sessions. The project team met monthly and conducted Plan-Do-Study-Act cycles periodically to address areas for improvement.

We encountered several implementation challenges, particularly early adoption of the EOS calculator—especially for newborns of mothers with chorioamnionitis—and consistent discontinuation of antibiotics at 36 hours when initiated. These barriers were addressed through five iterative PDSA cycles. Key interventions included development of a standardized EOS calculator smart phrase integrated into the admission history and physical template to prompt use and implementation of default 36-hour antibiotic stop dates requiring active modification to extend therapy, which improved timely discontinuation.

Implementation fidelity was assessed through periodic structured chart audits of eligible newborns during the intervention period. Audits evaluated documentation of EOS calculator use, concordance between recommendations and management, and adherence to the 36-hour antibiotic discontinuation protocol when cultures were negative. Fidelity was defined a priori as documented calculator use with management aligned with recommendations unless clinically justified, and results were reviewed with the clinical team during PDSA cycles to address gaps.

### Data collection, measures, and analysis

Data was prospectively collected starting in February 2023 for 12 months [post-EOS calculator time frame]. The primary outcome was the proportion of infants receiving antibiotics. Secondary outcomes included: NICU and hospital length of stay, duration of antibiotics, number of infants born to birthing parents with chorioamnionitis receiving antibiotics, and treatment for “culture-negative sepsis” (defined as a negative EOS blood culture treated with antibiotics for ≥5 days). Estimating cost saving in a NICU setting depends on NICU leveling and severity of illness, along with the fact that the origin of charges come from multiple areas. Cost savings were estimated using differences in NICU length of stay between the pre- and postintervention groups. Both direct hospital costs and direct patient charges were considered. To account for variability in NICU resource utilization, we calculated a range of estimated costs using per-day values for level II NICU care (lower-bound estimate) and level III NICU care (upper-bound estimate). The difference in NICU length of stay between groups was multiplied by the corresponding per-day cost or charge to estimate per-patient savings. These values were then extrapolated to estimate total savings per 1,000 NICU patients. Many infants, particularly in the post-EOS calculator time frame, would be transferred back to the general care nursery after an observation period in the NICU if they were not on antibiotics or after antibiotics were discontinued, which is why total hospital length of stay was used as a metric. Readmissions and the number of culture-positive EOS cases were tracked as balancing measures to ensure there were no unintended negative consequences associated with our interventions. Regarding process measure, documentation of Kaiser sepsis scores were tracked monthly for all eligible infants admitted to the NICU, as well as adherence to stopping antibiotics within 36 hours of obtaining blood culture if patients were not treated for culture-negative or culture-positive sepsis.

Categorical variables were analyzed using the *χ* ^2^ test, while continuous variables were compared using Student’s *t*-test or Wilcoxon rank sum. Bi- and multivariate analyses were performed with statistical significance defined as *P* value < .05. All analyses were performed using Stata SE version 19.0 (StataCorp, College Station, TX). To assess variation over time and the impact of the intervention longitudinally, run charts were used.

## Results

A total 1,306 infants were included: 814 in pre-EOS calculator cohort and 492 in the post-EOS calculator cohort. Baseline characteristics—including gestational age, birth weight, and percentage of vaginal deliveries—were similar between groups (Table 1). Seventy-two infants were excluded (42 pre-EOS, 30 post-EOS).

**Table 1.** Baseline characteristics

Variable	Pre-EOS calculator (*n* = 814)	Post-EOS calculator (*n* = 492)	*P*-value
Total Births	4,023	2,497	.60
Gestational age, in weeks	38 (34–41.71)	38 (34–42.43)	.41
Birth weight, in grams	2,910 (1370–5,740)	2,980 (1480–4,700)	.20
Male infants	423 (51.9)	245 (49.7)	.67
Vaginal deliveries	468 (57.5)	262 (53.3)	.14

Data are given as n (%), mean or median (IQR).

**Figure 2.**
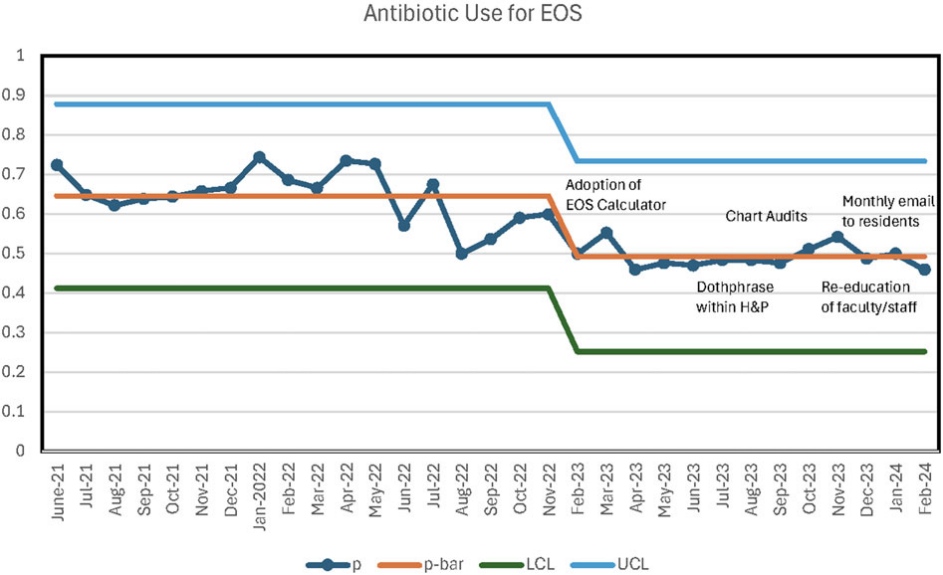
Antibiotic use for early-onset sepsis control chart.

Following the interventions, significant improvements were observed (Table 2). EOS antibiotic starts for infants ≥34 weeks’ gestation admitted to the NICU decreased from 62% (502/814) to 51% (252/492) (*P* < .01; OR of 0.6, 95% CI 0.47–0.75) (Figure 2), maintained after controlling for chorioamnionitis. In the chorioamnionitis subgroup, antibiotic starts decreased from 99% (151/152) to 50% (54/108) (*P* < .01). When started, antibiotic duration decreased from 48 to 33 hours, but this was not statistically significant. Days of antibiotic therapy per 1,000 NICU patient days decreased from 168 to 110 (*P* < .01), and total patient days decreased from 93 to 57 (*P* < .01). Fewer blood cultures were obtained (84% (685/814) vs. 67% (330/492), *P* < .01), and fewer complete blood counts per NICU patient were performed (*P* < .01), with this reduction observed in both infants on and off antibiotics. NICU admissions treated for culture-negative sepsis decreased from 7% (64/814) to 3% (16/492) (*P* < .01).

**Table 2.** Outcomes

Variable	Pre-EOS calculator (*n* = 814)	Post-EOS calculator (*n* = 492)	*P*-value
Number of patients antibiotics initiated on for EOS	508 (62.4)	252 (51.2)	**<.01**
Blood culture obtained	685 (84.2)	330 (67.1)	**<.01**
CBC obtained per patient (total)	2.84	1.69	**<.01**
CBC obtained per patient (on antibiotics)	3.54 (*n* = 508)	2.54 (*n* = 252)	**<.01**
CBC obtained per patient (No antibiotics)	1.58 (*n* = 306)	.76 (*n* = 240)	**<.01**
Positive blood cultures	4 (0.5)	0 (0)	.30
Antibiotics for CAM in the birthing parent	151 (99.3) ***n* = 152**	54 (50) ***N* = 108**	**<.01**
Duration of antibiotics, in hours	48 (40–48)	33 (31–36)	.12
Antibiotic DOT, in days			
Per 1,000 NICU patient days	168.6	110.1	**<.01**
Per 1,000 total patient days	93.8	57.3	**<.01**
Treatment for culture-negative sepsis	64 (7.9)	16 (3.3)	**<.01**
NICU LOS, in days	6 (3–11)	5 (2–10)	**<.01**
Hospital LOS, in days	6 (3–11)	5 (3–11)	**.01**
Readmissions	9 (1.1)	6 (1.2)	.852

LOS, length of stay, CAM, chorioamnionitis, CBC, complete blood count DOT, duration of therapy. Data are given as *n* (%), mean or median (IQR).

NICU length of stay decreased by a median of 1 day [6 days (IQR 3–11) vs 5 days (IQR 2–10)] (*P* < .01), maintained after controlling for chorioamnionitis and antibiotic use. Hospital LOS decreased by a median of 1 day [6 days (IQR 3–11) vs 5 days (IQR 3–11)] (*P* = .01) when controlling for gestational age. Estimated savings ranged from $916,000 to $1.84 million per 1,000 NICU patients in direct costs and $5.82 million to $12.5 million per 1,000 NICU patients in charges.

**Figure 3.**
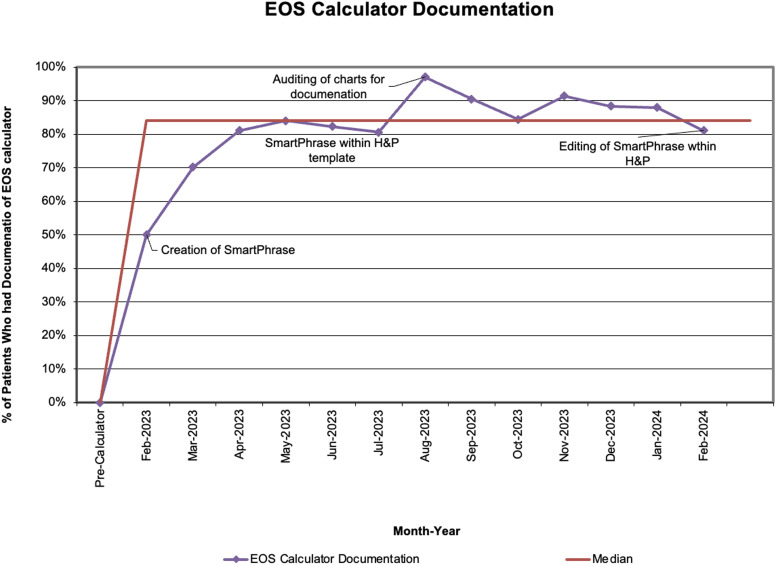
Early-onset sepsis calculator documentation run chart.

**Figure 4.**
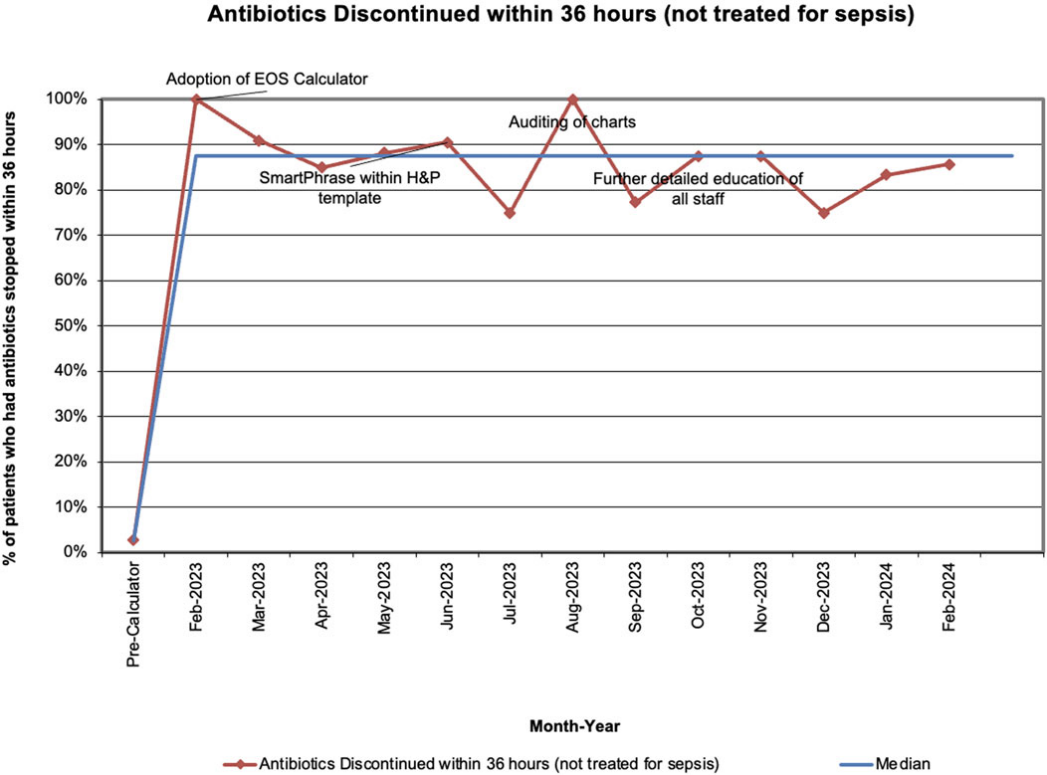
Antibiotics discontinued within 36 hours run chart (not treated for early-onset sepsis).

Documentation of EOS-calculator use met our goal (Figure 3), and adherence to stopping antibiotics within 36 hours of obtaining blood culture if patients were not treated for culture-negative or culture-positive sepsis significantly increased from 2.7% (22/814) to 85.5% (421/492) (Figure 4). There was no difference in readmissions (∼1% per group) or culture-positive EOS cases (4 (0.5%) vs 0).

## Discussion

Reduced exposure to antibiotics early in life is essential to avoid both short-term and long-term adverse effects. In this single-center quality improvement project, we demonstrated that the unnecessary utilization of antibiotics for risk of EOS decreases significantly with the use of an EOS calculator and limiting empiric antibiotic courses to 36 hours pending blood culture results. There were significant cost savings due to reduced LOS and resource utilization while admitted, and no negative consequences were identified due to the use of the EOS-calculator, including no missed cases of EOS nor increases in readmissions to the NICU. This is consistent with previous studies looking at the safety of the EOS-calculator, including a meta-analysis showing no increase in missed cases of EOS, overall EOS incidence, or readmissions.^26^ Similarly, we saw no negative consequences in decreasing empiric antibiotic use for EOS from 48 hours to 36 hours, which is consistent with other studies assessing decreasing empiric antibiotic time for EOS.^12^

There were two essential areas that contributed to a decrease in antibiotic use in our NICU. The first was the 50% reduction in antibiotic exposure to infants born to birthing parents with chorioamnionitis. Prior to implementation of this project, all infants born to birthing parents with chorioamnionitis were started on antibiotics. After the adoption of our standardized evaluation tool, antibiotics were only started for the highest-risk infants. The second was treatment for “culture-negative sepsis,” a common cited indication for antibiotics based on institutional review. Most studies suggest the diagnosis of “culture-negative sepsis” exceeds the incidence of culture-positive sepsis by up to 16-fold in the US,^27,28^ with “culture-negative sepsis” accounting for up to 20% of antibiotic use in the NICU.^27,29,30^ The reduction in “culture-negative sepsis” courses of antibiotics was partially due to the adoption of recommendations driven by the American Academy of Pediatrics that there is limited data supporting treating for sepsis based off laboratory findings alone [e.g. complete blood count, white blood cell count, *C*-reactive protein].^31–33^ However, an additional change accompanying institution of the EOS sepsis calculator was that less infants were empirically started on antibiotics, leading to less laboratory tests drawn and eventually to less treatment courses driven by laboratory findings alone. Even for infants who were started on empiric antibiotics, the quality improvement initiative increased awareness amongst providers on the benefits of limiting antibiotics, particularly for well-appearing infants, and providers were more willing to stop empiric antibiotics by 36 hours.

One difficult aspect of this project was getting buy-in from staff and changing of provider culture. There were previous failed discussions on integrating an EOS-sepsis calculator in the NICU, but with persistence and continued education, consensus was reached, leading to adoption of the EOS-sepsis calculator. The major cause of reluctance was fear of a missed EOS case. Data in large-scale studies shows no increased risk of missed cases or increased EOS incidence compared to other risk management strategies for EOS,^26,34^ but not every case of EOS is predictable, and clinical judgment remains very important to evaluation and treatment. To assess this risk, all cases of culture-positive EOS over the last 3 years at our institution were put into the EOS-calculator, and the calculator recommended treatment with antibiotics for all of cases, including one case where the clinical team had not started treatment until the culture returned positive. This demonstration was convincing to providers.

While the adoption of the EOS-sepsis calculator was the biggest contributor to the decrease in antibiotic utilization, the decrease in empiric antibiotics from 48 hours to 36 hours also contributed. This shift was driven by both internal data review and external studies, including a recent large study of EOS blood cultures showing 68% of pathogens grew within 24 hours and 94% grew within 36 hours of obtaining a blood culture.^35^

The goal of this initiative was to decrease unnecessary utilization of antibiotics for EOS, but one unique aspect of the study is the cost analysis associated with decreasing antibiotic use and subsequently decreasing NICU and hospital length of stay. This is consistent with recent studies looking at costs associated with the initiation of the EOS-calculator.^36,37^ There was a also cost savings, albeit smaller in gross amount, based on laboratory draws, including complete blood counts and cultures, as well as the costs of antibiotics.

This study has several limitations. With single-center study design, it is difficult to quantify how generalizable it is to other NICUs due to variation in antibiotic practice.^38^ While education was performed on the use of the EOS-calculator and documentation of the calculator were tracked, there was no assessment of whether the calculator and documentation were correct, outside of random audits of several charts a month. Also, while data shows adverse effects of over-use of antibiotics early in life, including increased late-onset sepsis and necrotizing enterocolitis,^13,14^ these parameters were not tracked in our project. While readmission rates were assessed, only readmissions to our own health system were viewable, and it is possible infants were admitted to other institutions.

The results of this study show improvement in antibiotic stewardship while also demonstrating further areas for improvement. This study is limited to inborn late preterm and full-term infants at risk for EOS; however, it was not focused on limiting antibiotics for outborn infants, premature infants, or infants at risk for late-onset sepsis. The next step in this initiative is improving antibiotic use in premature infants as the risks of antibiotic exposure particularly affect this population. After review of institutional data on antibiotic exposure to premature infants, both for EOS and late-onset sepsis, the next step will involve an evidence-based plan directing antibiotic starts and discontinuation of antibiotics for these high-risk infants. Additionally, with the updated Kaiser Permanente EOS-calculator^39^ there are plans to investigate the changes’ effect on our patient population. There is also a plan to continue assessing adherence and integration of the EOS-calculator into the electronic medical record. A future goal is limiting low-risk infant admissions to the NICU, particularly those who are well-appearing born to mothers with chorioamnionitis and allowing them to stay with their parents to continue bonding and enhance breastfeeding, if desired.

In conclusion, implementing a revised guideline centered on the EOS-calculator reduced antibiotic use from 62% to 51% in infants born at 34 weeks or greater admitted to the NICU. This was accompanied by significant reductions in NICU and hospital length of stay, leading to lower hospital costs and charges without any identified adverse effects.
