# Implementation of the Early-Onset Sepsis Calculator in a Tertiary Care NICU – CORRIGENDUM

**DOI:** 10.1017/ash.2026.10764

**Published:** 2026-07-27

**Authors:** Walid Hussain, Deborah Bondi, Pooja Shah, Elzbieta Kalata, Kelli Skvarenina, Simon Parzen-Johnson

The author’s name was incorrectly spelled in the article as originally published (Keli Skvarenina). This has been corrected to Kelli Skvarenina in the HTML and PDF versions of the article.

The authors apologise for the error and publish this notice for the scholarly record.
